# Dysregulated Interferon Response and Immune Hyperactivation in Severe COVID-19: Targeting STATs as a Novel Therapeutic Strategy

**DOI:** 10.3389/fimmu.2022.888897

**Published:** 2022-05-17

**Authors:** Mahdi Eskandarian Boroujeni, Agata Sekrecka, Aleksandra Antonczyk, Sanaz Hassani, Michal Sekrecki, Hanna Nowicka, Natalia Lopacinska, Arta Olya, Katarzyna Kluzek, Joanna Wesoly, Hans A. R. Bluyssen

**Affiliations:** ^1^ Laboratory of Human Molecular Genetics, Institute of Molecular Biology and Biotechnology, Faculty of Biology, Adam Mickiewicz University, Poznań, Poland; ^2^ Laboratory of High Throughput Technologies, Institute of Molecular Biology and Biotechnology, Faculty of Biology, Adam Mickiewicz University, Poznań, Poland

**Keywords:** COVID – 19, interferon response, immune hyperactivation, multiple organ damage, STAT signaling, Statinhib, therapy

## Abstract

A disease outbreak in December 2019, caused by a novel coronavirus SARS-CoV-2, was named COVID-19. SARS-CoV-2 infects cells from the upper and lower respiratory tract system and is transmitted by inhalation or contact with infected droplets. Common clinical symptoms include fatigue, fever, and cough, but also shortness of breath and lung abnormalities. Still, some 5% of SARS-CoV-2 infections progress to severe pneumonia and acute respiratory distress syndrome (ARDS), with pulmonary edema, acute kidney injury, and/or multiple organ failure as important consequences, which can lead to death. The innate immune system recognizes viral RNAs and triggers the expression of interferons (IFN). IFNs activate anti-viral effectors and components of the adaptive immune system by activating members of the STAT and IRF families that induce the expression of IFN-stimulated genes (ISG)s. Among other coronaviruses, such as Middle East respiratory syndrome coronavirus (MERS-CoV) and SARS-CoV, common strategies have been identified to antagonize IFN signaling. This typically coincides with hyperactive inflammatory host responses known as the “cytokine storm” that mediate severe lung damage. Likewise, SARS-CoV-2 infection combines a dysregulated IFN response with excessive production of inflammatory cytokines in the lungs. This excessive inflammatory response in the lungs is associated with the local recruitment of immune cells that create a pathogenic inflammatory loop. Together, it causes severe lung pathology, including ARDS, as well as damage to other vulnerable organs, like the heart, spleen, lymph nodes, and kidney, as well as the brain. This can rapidly progress to multiple organ exhaustion and correlates with a poor prognosis in COVID-19 patients. In this review, we focus on the crucial role of different types of IFN that underlies the progression of SARS-CoV-2 infection and leads to immune cell hyper-activation in the lungs, exuberant systemic inflammation, and multiple organ damage. Consequently, to protect from systemic inflammation, it will be critical to interfere with signaling cascades activated by IFNs and other inflammatory cytokines. Targeting members of the STAT family could therefore be proposed as a novel therapeutic strategy in patients with severe COVID-19.

## Introduction

Since the end of 2019, a novel coronavirus (SARS-CoV-2) caused a respiratory disease outbreak in Wuhan, that quickly spread in China and subsequently worldwide. Soon after that, the WHO declared SARS-CoV-2 infection a global health threat and named it coronavirus disease-19 (COVID-19). Clinically, COVID-19 varied from mild to severe symptoms, including acute respiratory distress syndrome (ARDS), as observed after SARS-CoV infection. As of February 2022, more than 350 million confirmed cases, including 5.6 million deaths have been reported.

SARS-CoV-2 is a SARS-CoV-like β-lineage coronavirus originating from bats, that initially infects cells from the upper and lower respiratory tract system through inhalation or contact with infected droplets (1, 2). Common clinical symptoms include fatigue, fever, and cough, but also shortness of breath and lung abnormalities. Still, some 5% of SARS-CoV-2 infections progress to severe pneumonia and ARDS, with pulmonary edema, acute kidney injury, and/or multiple organ failure as important consequences, which can lead to death (3–6).

The innate immune system recognizes viral RNAs *via* endosomal and cytosolic pattern recognition receptors, including TLRs or RIG-1 and MDA5. This triggers the expression of interferons (IFN), which are the mediators of cellular homeostatic responses to virus infection. IFNs comprise a family of antiviral cytokines, classified as type I, II, and III. Through binding to their cognate cellular receptors, they activate transcription factors of the STAT and IRF family and expression of IFN-stimulated genes (ISG)s, which turn on the anti-viral state and render cells more resistant to virus infection and thereby limit virus spread (7, 8). Among other coronaviruses, such as Middle East respiratory syndrome coronavirus (MERS-CoV) and SARS-CoV, common strategies have been identified to antagonize IFN signaling. This typically coincides with hyperactive inflammatory host responses known as the “cytokine storm” that mediate severe lung damage.

Based on recent COVID-19 patient and animal studies it can be concluded that early during SARS-CoV-2 infection of the upper airways a dysregulated IFN response, mediated by antagonizing IFN induced signaling pathways, is a hallmark of SARS-CoV-2 infections. Later in the lungs, this is accompanied by the production of inflammatory cytokines, together with excessive IFN production, known as the “cytokine storm”. The excessive inflammatory response in the lungs is associated with the local recruitment of immune cells, including macrophages, effector T-cells, dendritic cells, and neutrophils that create a pathogenic inflammatory loop (9, 10). Together, it causes severe lung pathology, including ARDS, as well as damage to other vulnerable organs, like the heart, spleen, lymph nodes, and kidney, as well as the brain. This can rapidly progress to multiple organ exhaustion and correlates with a poor prognosis in COVID-19 patients (6, 9, 10).

So far, several effective vaccines have been generated to prevent infection and transmission. On the other hand, efforts to increase our understanding of the biology of SARS-CoV-2 infection and the host immune response are urgently pursued to design therapeutic strategies that reduce the risk of severe COVID-19 and the overall mortality rate.

## SARS-CoV-2 Infection, Exuberant Inflammation, and Clinical Outcome

SARS-CoV-2 infection starts with its entrance into human cells. Like SARS-CoV, SARS-CoV-2 engages angiotensin-converting enzyme-2 (ACE2) as a cellular entry receptor. In addition, it uses the cellular serine protease TMPRSS2 for S protein priming, whereas Neuropilin1 (NRP1) serves as a co-factor in cell entry (11–14). Thus, effective SARS-CoV-2 transmission requires high co-expression of ACE2 and TMPRSS2 in target cells, including nasal epithelial cells and type II alveolar cells (AT2) of the lungs (15). Viral-Track was developed as a new computational pipeline, to detect viral RNA in single-cell transcriptome data of bronchoalveolar lavage fluid (BALF) from COVID-19 patients. Detecting viral RNA only in severe disease (16), particularly in ciliated and epithelial progenitors and in a subset of macrophages (SPP1+ macrophages), suggested differential progression of infection in the lung (16, 17).

In addition to the nose and lung, the extrapulmonary spread of SARS-CoV-2 was observed (13). Interestingly, Zou et al. recently analyzed single-cell RNA sequencing datasets combined with ACE2 expression and identified potential risk organs, including the lung, heart, kidney, ileum, esophagus, and bladder (18). At the same time, specific cell types could be located (i.e., ileum and esophagus epithelial cells, bladder urothelial cells, AT2, myocardial cells, and proximal tubule cells of the kidney), being sensitive to SARS-CoV-2 infection (19). In fact, ACE2 distribution in the host organism importantly correlated with organ injury. Likewise, expression differences and/or ACE2 polymorphisms determine SARS-CoV-2 infection susceptibility and clinical manifestations. As part of the renin-angiotensin-aldosterone system (RAAS), ACE2 converts angiotensin II (Ang II) to Ang (1–7). Therefore, ACE2 has a dual role in both SARS-CoV and SARS-CoV-2 infection. On the one hand, it acts as the host cell virus entry receptor, but on the other by protecting the lungs and other organs from Ang II-mediated vasoconstrictive, pro-inflammatory, and pro-fibrotic injury on pulmonary vascular and epithelial cells (6, 20).

Common clinical symptoms of SARS-CoV-2 infection include fatigue, fever, and cough, but also shortness of breath and lung abnormalities. A mere 20% of COVID-19 patients require hospitalization and about 1 in 4 of these progress to severe pneumonia and ARDS, with pulmonary edema, acute kidney injury, and/or multiple organ failure as important consequences, that require invasive mechanical ventilation (3–5). In common with SARS-CoV and MERS-CoV infections, ARDS causes similar immunopathogenic features and is the main cause of death in COVID-19 disease (4). Advanced age and the presence of certain comorbidities, such as coronary heart disease, hypertension, chronic obstructive pulmonary disease, and diabetes mellitus, are among the risk factors for the development of severe COVID-19 and poor outcome (3, 21–25).

An important hallmark of ARDS is the cytokine storm - an excessive inflammatory response of immune effector cells combined with the release of pro-inflammatory cytokines and chemokines (4). Indeed, SARS-CoV-2 infected individuals with severe symptoms display increased serum concentrations of many pro-inflammatory mediators, including interferon (IFN)-g-induced protein 10 (IP-10), monocyte chemoattractant protein 1 (MCP1), macrophage inflammatory protein (MIP)-1a, platelet-derived growth factor (PDGF), TNF-a, vascular endothelial growth factor (VEGF), granulocyte colony-stimulating factor (G-CFS), IL-1b, IL-2, IL-6, IL-7, IL-8 (4, 26–29). Also, recent single-cell expression profiling studies in bronchoalveolar cells, PBMCs, and airway epithelium from COVID-19 patients, have shown that upregulation of pro-inflammatory pathways correlates with severe symptoms (17, 30). Therefore, an excessive inflammatory response, associated with high levels of circulating pro-inflammatory cytokines and chemokines, profound lymphopenia, and substantial infiltration of hyper-active mononuclear cells in the lungs, is a major cause of disease severity and death in patients with COVID-19. In addition, it has also become clear that SARS-CoV-2 infected individuals can rapidly progress to a multiple organ dysfunction syndrome (MODS). In this respect, increased fibrin degradation products (identified as D- dimers) and coagulation abnormalities (i.e, apparent thrombotic manifestations and prolonged prothrombin time) as well as lower platelets counts, promote vascular leakage and disseminated intravascular coagulation. These vascular abnormalities could lead to organ failure and death and are a clear link with poor prognosis in patients with severe COVID-19 (3–5, 31, 32).

## SARS-CoV-2 Infection and Dysregulated IFN Responses

The innate immune system recognizes viral RNAs *via* endosomal and cytosolic pattern recognition receptors, including Toll-like receptor (TLR) 3 and TLR7 or retinoic acid–inducible gene I (RIG-I) and melanoma differentiation-associated protein 5 (MDA5) (33, 34). After infection of the target cells, they trigger the activation of the downstream transcription factors, namely interferon regulatory factor (IRF)3 and IRF7 and nuclear factor-kappa B (NF-κB). These factors collectively induce the production of IFNs, the main mediators of cellular homeostatic responses to virus infection (7, 8).

IFNs consist of three sub-types, including IFN-I, IFN-II, and IFN-III. IFN-II, also known as IFNγ, mediates broad immune responses to pathogens by binding to the IFNγ receptor (IFNGR) complex. In response to foreign antigens or mitogens, IFN-II is mainly produced by T lymphocytes and Natural Killer (NK) cells, to modulate adaptive immunity as well as inflammation. Although IFN-II exhibits anti-viral activity, IFN-I is the main anti-viral IFN subtype, predominantly consisting of IFNα and IFNβ subtypes. IFN-I can be produced by many cell types and collectively bind to the IFNα receptor (IFNAR) to potently inhibit viral infection as part of a robust innate immune response. IFN-III (consisting of IFNΛ1 to -4 subtypes) is largely produced by epithelial cells, hepatocytes, and dendritic cells, in a tissue-specific manner. All IFNΛ subtypes bind the heterodimeric IFN-Λ receptor (IFNLR) (35), which is restricted to certain cell types and tissues, including macrophages, conventional dendritic cells (DCs), and plasmacytoid dendritic cells (pDCs), neutrophils, and respiratory epithelial cells. IFN-Λ responses, therefore, confer more localized antiviral protection at the site of infection. In addition to antiviral and pro-inflammatory activity, IFNs exert anti-proliferative and pro-apoptotic functions (7, 8).

As potent IFN-producing cells also plasmacytoid (p)DCs are important cellular mediators of the antiviral response by producing massive amounts of IFN-I and IFN-III. In addition, they produce other pro-inflammatory cytokines, which modulate the function of innate and adaptive immune cells (35).

Coronaviruses, such as SARS-CoV and MERS-CoV, have developed strategies to antagonize IFN responses. This typically coincides with hyperactive inflammatory host responses that lead to severe lung damage. Using mice infected with SARS-CoV, it was shown that robust virus replication accompanied by delayed IFN-I signaling triggers hyperinflammatory and severe immune pathology of the lungs as well as reduced survival. This dysregulated IFN-I signaling promoted the infiltration of pathogenic inflammatory monocyte macrophages (IMM), leading to increased lung cytokine/chemokine levels, and resulting in vascular leakage and impaired virus-specific T cell responses (30). Likewise, robust and persistent expression of IFN and IFN-stimulated genes (ISGs) in association with impaired T cell and antibody responses was observed in fatal SARS in humans (36). Thus, during SARS-CoV and MERS-CoV infection, an ineffective IFN-I response, accompanied by excessive production of inflammatory cytokines, correlates with disease severity and poor prognosis and appears to be a hallmark of coronavirus infections (37–39).

**Figure 1:**
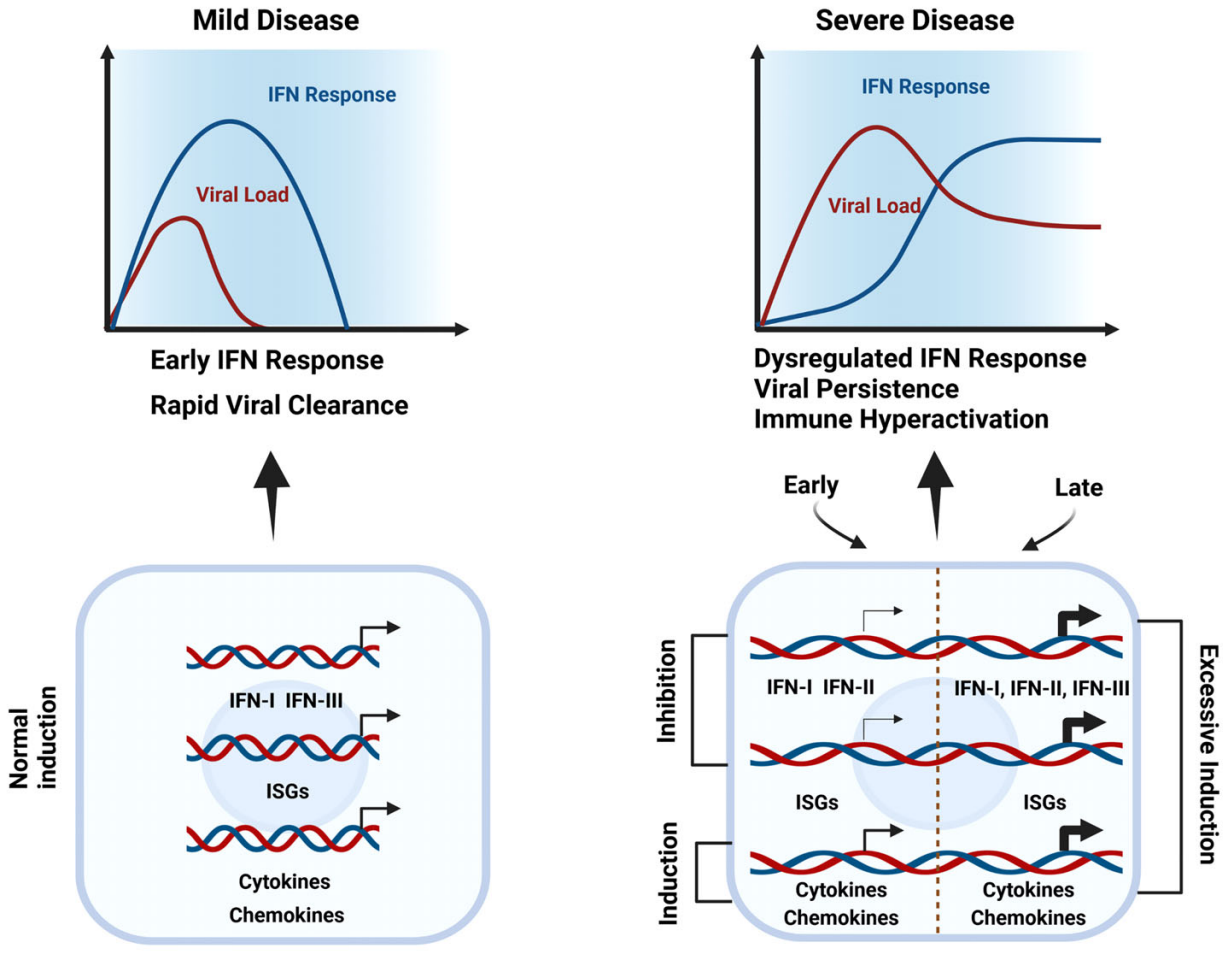
Dysregulated IFN responses & immune hyper-activation in severe COVID-19. During mild disease, the initial viral burden is low and IFNs, together with cytokines, chemokines, and other ISGs are induced early and rapidly to mediate effective viral clearance (left). In severe COVID-19 (right), in the upper respiratory tract interferon production and ISG expression are inhibited by SARS-CoV-2, diminishing the immune response and promoting virus survival (Early). But by the time the virus enters the lower respiratory tract, an exuberant immune response is activated, including excessive upregulation of interferons, cytokines, chemokines, and other ISGs that at this point are harmful (Late). Thus, both early-stage deficiencies in type I IFN responses and late-stage IFN hyper-responsiveness are clear characteristics of severe COVID-19.

As stated by Zhang et al: “Combined immunodeficiencies, immune dysregulation disorders and defects of innate immunity impairing type I interferon responses show a higher rate of severe disease and higher mortality. In addition, inborn errors of type I interferon responses and inborn errors of immunity in which patients harbor autoantibodies against these cytokines, confer a high risk for critical COVID-19” (40). Together, this reflects the key role of IFN-I in the host defense against SARS-CoV-2 (**Figure 1**). Using peripheral blood mononuclear cells (PBMCs), Lee et al. (30) performed single-cell RNA-seq to identify driving factors of COVID-19 progression. By comparing healthy donors, patients with mild or severe COVID-19, and patients with severe influenza, they observed that all PBMC cell types of COVID-19 displayed hyper-inflammatory signatures, marked by a TNF/IL-1β-mediated inflammatory response, as compared to severe influenza. Moreover, classical monocytes from severe COVID-19 patients displayed a type I IFN response together with the TNF/IL-1β-mediated inflammation, being absent in mild COVID-19 patients. Remarkably, patients with severe influenza also presented these type I IFN-dependent inflammatory features. Consequently, it was proposed that the type I IFN response plays an important role in the excessive inflammation seen in severe COVID-19 patients (30) (**Figure 1**).

Conversely, by studying peripheral blood responses of COVID-19 patients, Hadjadj et al. (41) observed reduced IFN responses in critically ill patients paired with a pro-inflammatory response. By comparing the transcriptional responses of several respiratory viruses, including SARS-CoV-2, Blanco-Melo et al. demonstrated *in vitro* that the host response to SARS-CoV-2 is unable to activate a robust IFN-I and -III response whereas high levels of chemokines were presented for the recruitment of immune cells (42). Along the same lines, Broggi and colleagues evaluated the ability of SARS-CoV-2 to induce IFNs in the upper or lower airways of COVID-19 patients. Importantly, they observed that IFN levels in the upper respiratory tract of COVID-19 patients did not significantly differ from healthy individuals. But bronchoalveolar lavage fluid from these patients exhibited increased levels of inflammatory cytokines, IFN-I, and IFN-III. This indicated that in the upper airways interferon production is inhibited by SARS-CoV-2, diminishing the immune response and promoting virus survival (**Figure 1**). But by the time the virus enters the lower respiratory tract, an excessive immune response is activated, together with upregulation of interferons that at this point is harmful. In conclusion, both early-stage deficiencies in type I IFN responses and late stage IFN hyper-responsiveness are a characteristic of severe COVID-19 (43, 44). Indeed, an early transient response of IFNa and IFNΛ was observed in mild-to-moderate COVID-19, whereas in severe patients, levels further enhanced towards the second week (45). The longitudinal dynamic changes of IFN-I production in severe patients correlate with experiments in a mouse SARS-CoV-2 model, further proving *in vivo* that IFN-I is unable to control SARS-CoV-2 replication but importantly drives hyperactive immune responses (46).

Therefore, it is tempting to propose that during early SARS-CoV-2 infection of the upper respiratory tract, intervention with recombinant interferons and other antivirals offer important solutions. However, during hyper-inflammation in the lower respiratory tract, it will be critical to interfere with signaling cascades activated by IFNs and other inflammatory cytokines, probably with anti-inflammatory drugs.

## SARS-CoV-2 Evolved Strategies to Target IFN Signaling

**Figure 2:**
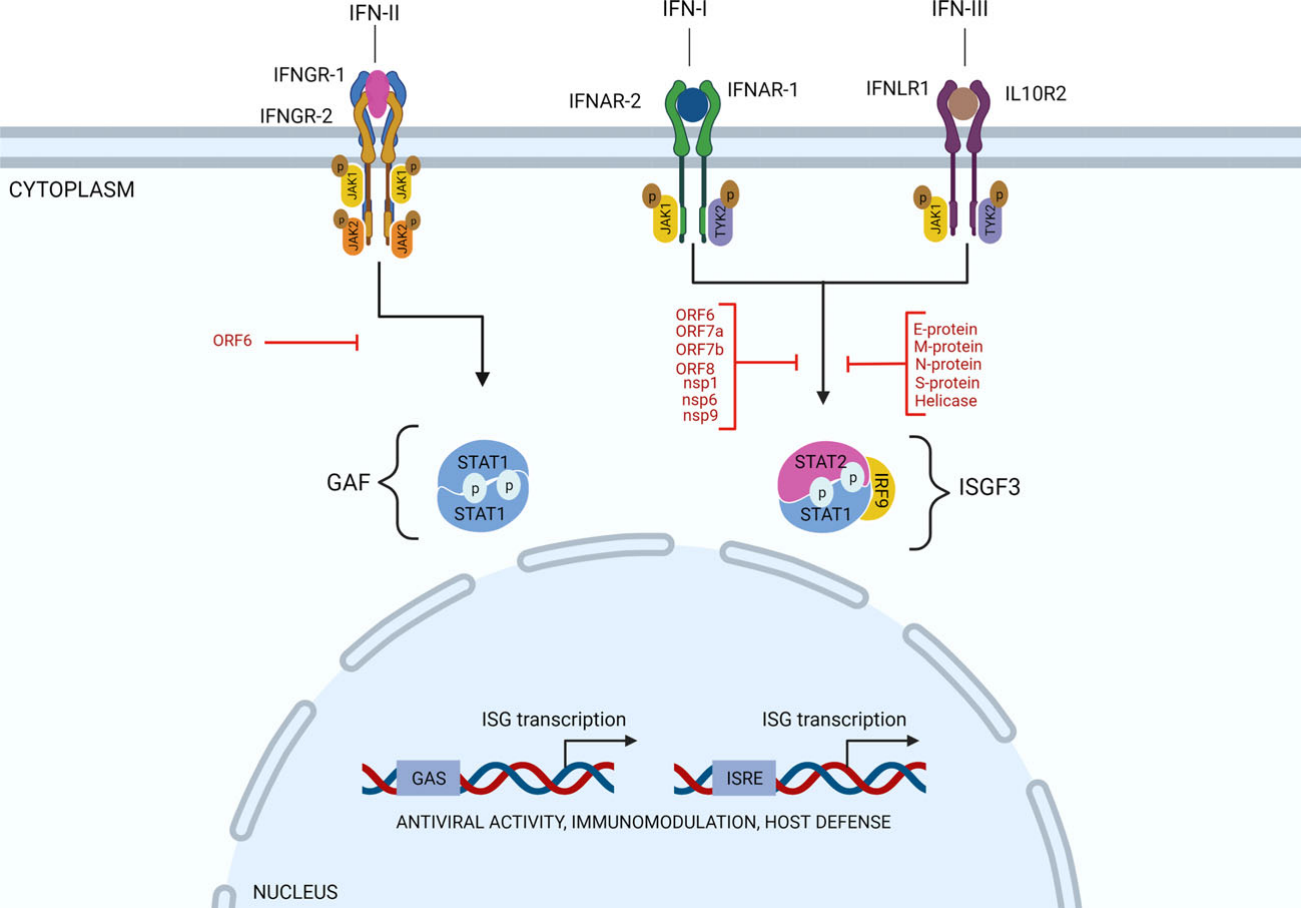
SARS-CoV-2 evolved strategies to target IFN signaling. Through binding to their cognate cellular receptors and Janus kinase (JAK)-dependent phosphorylation, IFNs activate transcription factors of the Signal Transducer and Activator of Transcription (STAT) family and expression of IFN-stimulated genes (ISG)s. Thus, IFN-I, IFN-II, and IFN-III induce ISG expression by phosphorylating STAT1 and/or STAT2. IFN-II-induced STAT1 homodimers regulate gene expression of IFN-II activation site (GAS) containing genes. The association of IRF9 with STAT1/2 heterodimers (known as ISGF3), expands the range of enhancer elements targeted by IFN-I and IFN-III to IFN-stimulated response element (ISRE). Various SARS-CoV-2 proteins, as indicated (in red), have been identified to inhibit the IFN response. Particularly, by interfering with STAT1 and/or STAT2 activation and ISG expression, as explained in the text.

As mentioned above, after infection of target cells, RNA viruses trigger the activation of downstream IRF3 and IRF7 and NF-κB transcription factors and the production of IFNs. Subsequently, through binding to their cognate cellular receptors and Janus kinase (JAK)-dependent phosphorylation, IFNs activate transcription factors of the Signal Transducer and Activator of Transcription (STAT) family and IFN-stimulated gene (ISG) expression, which turn on the anti-viral state and render cells more resistant to virus infection and thereby limits virus spread. Thus, IFN-I, IFN-II, and IFN-III induce ISG expression by phosphorylating STAT1 and/or STAT2. IFN-II-induced STAT1 homodimers regulate gene expression of IFN-II activation site (GAS) containing genes. The association of IRF9 with STAT1/2 heterodimers (known as ISGF3), expands the range of enhancer elements targeted by IFN-I and IFN-III to IFN-stimulated response element (ISRE) (**Figure 2**). Similarly, IRF family members, including IRF1, IRF7 and IRF8, can regulate ISG expression in response to all three types of IFN by binding the ISRE. Together, this leads to the transcriptional activation of hundreds of ISGs, such as MX1 and 2, ISG15, IFIT1, IFIT2 and IFIT3, and OAS1, OAS2, and OAS3 that importantly participate in the antiviral response (7, 8).

Among coronaviruses, such as MERS-CoV and SARS-CoV, common strategies have been identified to antagonize IFN production and inhibit IFN signaling (39, 47, 48). For example, SARS-CoV was shown to antagonize the IFN response using nsp1, papain-like protease (PLpro), nsp7, nsp15, ORF3b, M, ORF6, and N proteins (49, 50). Thus, several antagonizing mechanisms have been identified, including inhibition of IRF3 and IRF7 activation, blocking expression of STAT1, and interaction with ISG15.

Concerning SARS-CoV-2 infection and dysregulated IFN-I responses, likewise, nsp1, nsp12, nsp13, nsp14, and, nsp15 and orf6 and the M protein (51–53) were among potent IFN-I inhibitors. In addition, Li et al. observed that ORF6, ORF8, and nucleocapsid proteins significantly inhibited IFN-I production, and the IFN-I-mediated innate immune response (53). The same was true for ORF3b (54). A more detailed functional analysis of these proteins in relation to COVID-19 progression was performed by Xia et al. Importantly, they found that nsp6 suppressed IRF3 phosphorylation by binding TANK binding kinase 1 (TBK1), nsp13 blocked and bound TBK1 phosphorylation, and ORF6 inhibited IRF3 nuclear translocation by binding importin Karyopherin a 2 (KPNA2). They also identified nsp1 and nsp6 to block IFN-I signaling by interfering with the phosphorylation of STAT1/STAT2 or their nuclear translocation (**Figure 2**). Interestingly, nsp1 and nsp6 proteins of SARS-CoV-2 antagonized IFN-I signaling more efficiently than the counterparts of SARS-CoV and MERS-CoV suggesting that SARS-CoV and SARS-CoV-2 use different mechanisms to affect disease onset and progression (55). Along the same lines, Zhang et al. observed that the SARS-CoV-2 M protein did not significantly affect IRF3 phosphorylation, but blocked its nuclear translocation. Likewise, the S protein was found to suppress STAT1 phosphorylation and nuclear translocation by intervening with JAK1-STAT1 interaction (56). On the other hand, without directly influencing STAT1 and STAT2 activation, nsp7 and ORF7a clearly inhibited ISRE promoter activity (56). Nsp9 and ORF8 exhibited inhibition of STAT1 activation, whereas Helicase and E protein intervened with STAT1 and STAT2 activation (56). The same was shown by Shi et al. with the M protein significantly blocking STAT1 phosphorylation and ISG expression (55). This study by Shi et al. also provided evidence to suggest that ORF7a inhibited STAT2 phosphorylation, whereas ORF7b inhibited phosphorylation of both STAT1 and STAT2 (55). In a separate study, Mu et al. found that SARS-CoV-2 replication was efficiently enhanced upon N protein overexpression in infected human cells, by interacting with STAT1 and STAT2 and inhibiting their phosphorylation and nuclear translocation (57). (**Figure 2**). Finally, the S protein also interfered with IFN-I signaling, however with a currently unknown mechanism (51, 57).

Analysis of gene expression profiles from COVID-19 patient airway epithelial cells together with epithelial cell lines infected with SARS-CoV-2 revealed that also IFN-II signaling is antagonized in a SARS-CoV-2 dependent manner (58). Particularly, it was shown that the SARS-CoV-2 ORF6 protein suppressed NLRC5, an MHC class I trans-activator, both transcriptionally and functionally (**Figure 2**). This was mediated by blocking type II interferon-mediated STAT1 signaling, and subsequent NLRC5 and IRF1 gene expression. At the same time, ORF6 was shown to inhibit NLRC5 nuclear import, uncovering a novel immune evasion mechanism toward modulation of the MHC class I pathway.

Finally, it was assessed how JAK/STAT signaling was altered in immune cells in COVID-19 patients and if an imbalance in JAK/STAT signaling contributed to disease severity. Rincon-Arevalo et al. reported increased expression in mild and severe COVID-19 patients of STAT1 and IRF9, in correlation with peripheral monocytes exhibiting IFN signatures [marked by Siglec-1 [CD169] expression (59)]. Remarkably, CD14+ monocytes and plasmablasts in severe patients displayed reduced expression of STAT1 and Siglec-1 in comparison to mild patients. In contrast, STAT1 phosphorylation was increased in severe COVID-19 cases, pointing to an imbalanced JAK/STAT signaling and lack of ISG induced transcription. This defect was still present in PBMCs isolated from severe COVID-19 patients stimulated with IFN-α and IFN-γ. This strongly implies that impaired JAK/STAT signaling could serve as a potential predictive biomarker of severe COVID-19 in combination with patient stratification and IFN response targeted therapy.

Together a picture emerges in which multiple components of the type I, II and III IFN systems are targeted by SARS-CoV-2, including members of the STAT family (**Figure 2**) and also IRFs. This affects important aspects of the innate and adaptive immune system and leads to a dysregulated immune response.

## IFNs and Immune Cell Hyperactivation in Lung Pathology of Severe COVID-19

As with SARS-CoV and MERS-CoV infected patients, acute pneumonia is the most frequent and critical clinical manifestation presented among severe COVID-19 patients (2–6). Severe lung pathology after MERS-CoV and SARS-CoV infection is a consequence of dysregulated IFN-I responses and rapid virus replication, which promote the accumulation of pathogenic inflammatory monocyte macrophages (IMM) and IFN-producing pDCs, and the release of elevated lung cytokine/chemokine levels. Subsequently increased influx of IMM resulted in exuberant inflammation. In addition, T cell responses are reduced through IFN-I-mediated T cell apoptosis. Together, this causes severe lung pathology that correlated with poor outcome of patients infected with MERS-CoV and SARS-CoV (60–63).

**Figure 3:**
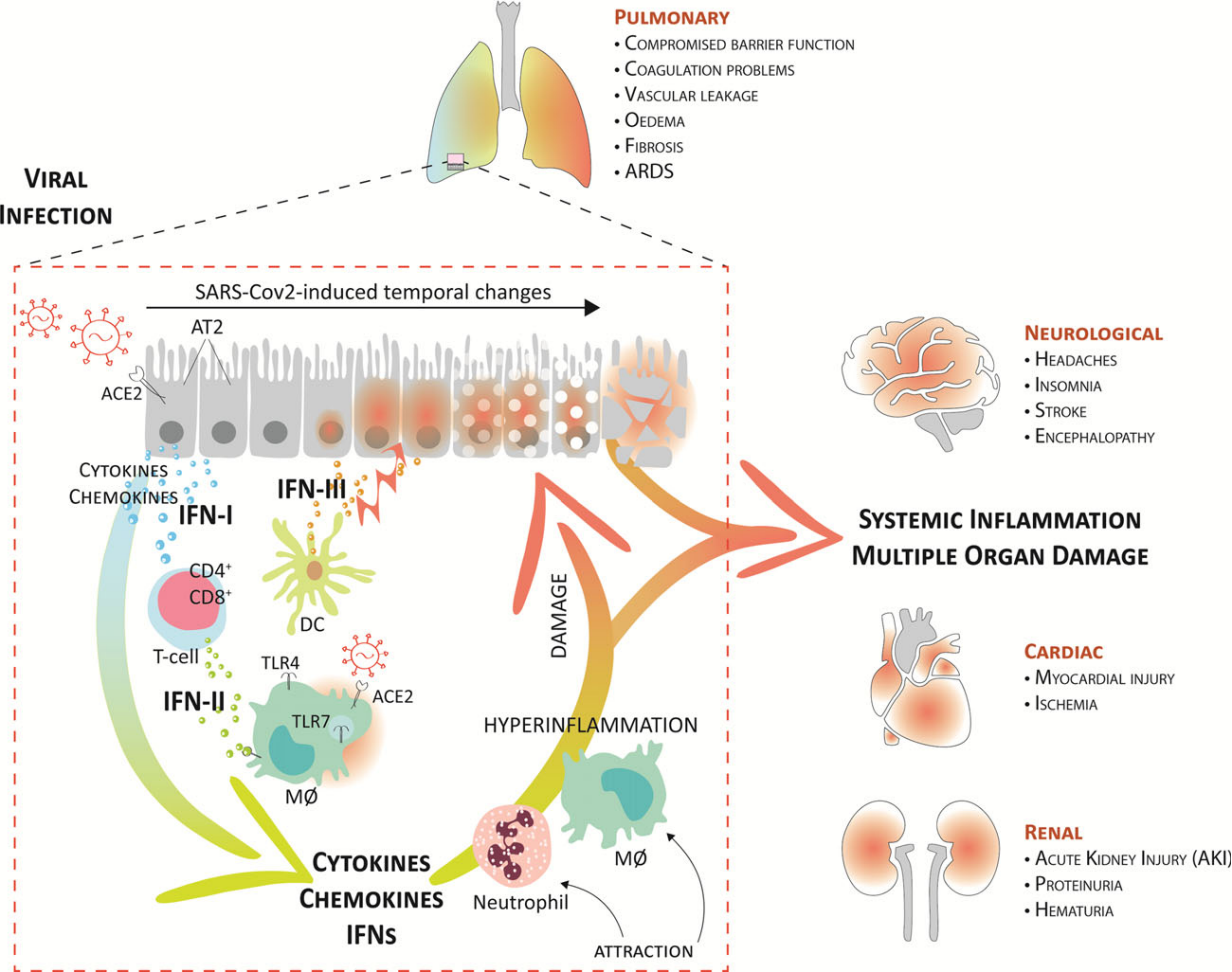
Systemic inflammation and multiple organ damage in severe COVID-19. A current model suggests that upon upper airway entry, SARS-CoV-2 impregnates the lower lungs *via* ACE2, where it infects several target cells, including the alveolar type 2 cells (AT2), alveolar macrophages (MØ), and vascular endothelial cells. Virus cell entry recognition *via* endosomal and cytosolic pattern recognition receptors (as TLR3 and TLR7) trigger the production of IFNs and proinflammatory mediators. Together, the local recruitment of effector T-cells (CD4+ and CD8+) and dendritic cells (DCs), further perpetuates higher proinflammatory cytokines, IFNs, and chemokines in the lung fluid. Consequently, this further augments damage to the lung tissue, mediated by endothelial dysfunction and vasodilation, and leads to the attraction of more inflammatory neutrophils and macrophages that create a pathogenic inflammatory loop in the lung. On the one hand, this leads to respiratory/organ failure and on the other hand, it also results in a “systemic cytokine storm” that causes widespread inflammation and damage to other vulnerable organs, like the heart, kidney, and brain. This can rapidly progress to multiple organ exhaustion, which correlates with a poor prognosis in COVID-19 patients.

Likewise, SARS-CoV-2 infection combines a dysregulated IFN response with excessive production of inflammatory cytokines in the lungs. In COVID-19 patients, this also has proven to be a main cause of disease severity and death and is associated with high levels of circulating pro-inflammatory cytokines and chemokines and the local recruitment of immune cells (6, 10, 64, 65). A current model suggests that upon upper airway entry, SARS-CoV-2 impregnates the lower lungs, where it infects a number of target cells, including the alveolar type 2 cells (AT2), alveolar macrophages, and vascular endothelial cells (**Figure 3**). Virus cell entry recognition *via* endosomal and cytosolic pattern recognition receptors (as TLR3 and TLR7) triggers the activation of downstream IRF3 and IRF7 and NF-κB transcription factors and the production of IFNs and proinflammatory mediators. Severe COVID-19 also displays increased CD4+ and CD8+ T cell activation. Yet, with the observed lymphopenia in these patients it is anticipated that in response to the virus, effector T cells are recruited to the lung. In this respect, lymphopenia has been recognized as a hallmark of severe COVID-19 (4, 5, 10), whereas an elevated neutrophil/lymphocyte ratio has been shown to serve as a biomarker of disease severity (65–68). Interestingly, plasmacytoid DC (pDCs), the main source of IFN-I and IFN-III, were also reduced in abundance in the circulation (65, 69).

Together, the local recruitment of immune cells further perpetuates proinflammatory cytokine and chemokine expression in BALF (CCL2, CCL3, CCL4, and CXCL10) but also in the circulation (IL-1, IFN-I, IFN-II, and IFN-III, IL-17, TNF, IP-10, MCP-1, G-CSF, GM-CSF, IL-1RA, CCL2, CCL3, CCL5, CCL8, CXCL2, CXCL8, CXCL9, and CXCL16) (4–6, 41, 42, 64, 70–72). Consequently, this further augments damage to the lung tissue, mediated by endothelial dysfunction and vasodilation, and leads to the attraction of more inflammatory neutrophils and macrophages that create a pathogenic inflammatory loop in the lung (**Figure 3**). These systemic cytokine profiles show overlap with cytokine release syndromes, and this highly suggests that COVID-19-associated hyper-inflammation is mediated by an over-activated mononuclear phagocyte (MNP) compartment (73, 74).

Interestingly, excessive IFN production, especially IFN-III, has shown to be an important factor impairing lung epithelial regeneration. IFN-III, unlike other IFNs, activates antiviral response and at the same time limits tissue-damaging functions of neutrophils. Major et al. (75) and Broggi et al. (43) showed that both IFN-I and IFN-III, produced by lung-resident dendritic cells through TLR-3 stimulation, disrupt lung epithelial cell repair during influenza recovery in mice in lower airways. IFN-λ is prevalent compared to IFN-I in virus-infected lungs. Specifically, IFN-λ directly affects epithelial cell proliferation and viability to inhibit tissue repair. With the presence of IFN-I and IFN-III in the lower, but not upper, airways of patients with COVID-19, it is therefore proposed that also during SARS-CoV-2 infection, especially IFN-III produced by activated DCs, acts on lung epithelial cells to compromise lung barrier function (76, 77) (**Figure 3**).

Recently, the cyclic GMP-AMP synthase (cGAS)–stimulator of interferon genes (STING) pathway was implicated in the pathological type I IFN responses in COVID-19. Indeed, a STING-dependent type I IFN signature was observed primarily in macrophages adjacent to areas of endothelial cell damage of COVID-19 skin manifestations. Under these conditions, SARS-CoV-2 infection activated cGAS–STING signaling in endothelial cells through mitochondrial DNA release, resulting in cell death and type I IFN production. Likewise, in lung samples from patients with COVID-19 with prominent tissue destruction, cGAS–STING activity was associated with type I IFN responses. Finally, in SARS-CoV-2 infected epithelial cell lines, such as Calu-3 and A549-ACE2, cGAS–STING activation was also linked to the production of pro-inflammatory cytokines. Together, this potentially identified the cGAS–STING pathway, activated by damaged mitochondrial DNA and not SARS-CoV-2, as a major driver of pathological type I IFN and pro-inflammatory cytokine responses in severe COVID-19 (78–80).

The excessive infiltration of inflammatory immune cells and the amplified production of cytokines, chemokines, and IFNs, observed in severe COVID-19, result in vascular leakage, coagulation abnormalities, and diminished lung barrier function, which stimulate endotheliitis and lung edema. This limits oxygen exchange and promotes hypoxia, leading to lung failure (81, 82). On the other hand, these excessive inflammatory conditions also result in a “systemic cytokine storm” that causes widespread inflammation and damage to other vulnerable organs, like the heart, spleen, lymph nodes, and kidney, as well as the brain. This can rapidly progress to multiple organ exhaustion, which correlates with a poor prognosis in COVID-19 patients (6, 9, 10) (**Figure 3**).

**Figure 4:**
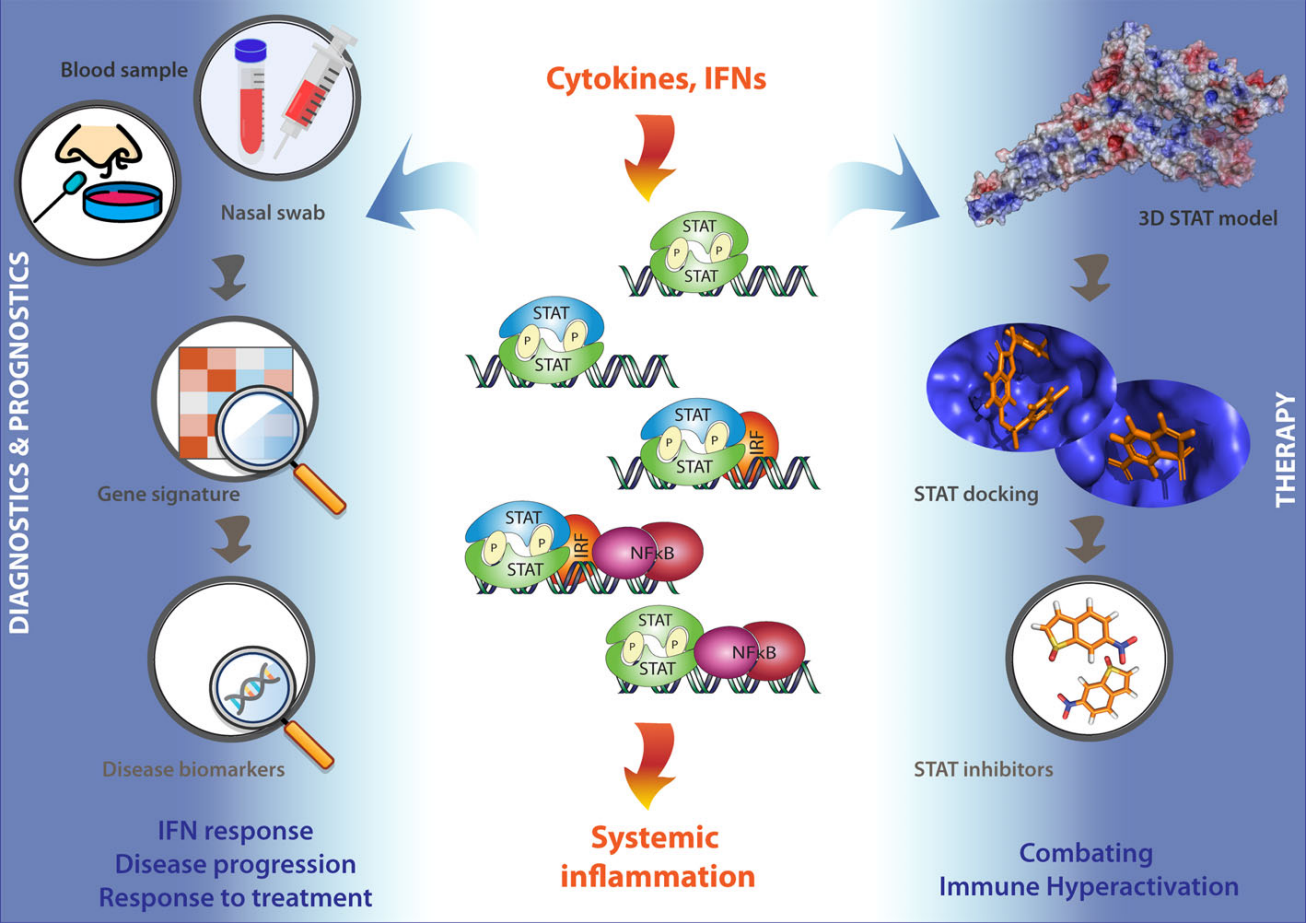
STATs as potential therapeutic targets in COVID-19 patients. Under conditions of excessive inflammation, maximum expression of many pro-inflammatory cytokine and IFN-stimulated genes has shown to depend on combinatorial actions of transcription factors of the STAT, IRF, and NF-kB families. This identifies STATs as potential therapeutic targets in severe COVID-19. Thus, a STAT-dependent pro-inflammatory gene signature could serve as a novel diagnostic tool to monitor and diagnose IFN responses, disease progression, and responses to treatment using blood or nasal swabs from COVID-19 patients (Left). On the other hand, the further testing and optimizing of already available Statinhibs, together with the identification of new Statinhibs using *in silico* 3D STAT modeling, virtual docking and screening, and *in vitro* validation, may offer new clinical benefits in combating immune hyper-activation during severe COVID-19 (Right).

It is important to realize that under these COVID-19 associated excessive inflammatory conditions, immune cell activation and cytokine and IFN release is tightly regulated by IFN and TLR signaling. Accordingly, maximum expression of many of these pro-inflammatory cytokines and IFN-stimulated genes has shown to depend on combinatorial actions of transcription factors of the STAT, IRF, and NF-kB families. This identifies STATs as potential therapeutic targets in severe COVID-19 (83–88) (**Figure 4**).

Further evidence for this was proposed in one publication where hyperactivation of STAT3 was linked to COVID-19-associated coagulopathy and lung damage. Specifically, plasminogen activator inhibitor-1 (PAI-1) upregulation, as an important STAT3 target, was linked to coagulopathy. TLR4 bound PAI-1 on macrophages could also induce the local production of proinflammatory cytokines and chemokines. This leads to the influx of active innate immune cells to the SARS-CoV-2 infected lung and drives the destruction of lung architecture. Based on this, STAT3 inhibition was proposed as a treatment strategy for COVID- 19 (89). In another study, in Syrian hamsters, which are highly sensitive to SARS-CoV-2 infection and virus-induced lung pathology (90), a STAT2-mediated dysregulated immune response was identified as the driving force. In particular STAT2-dependent type I and III IFN signaling was shown to restrict infection and systemic dissemination of SARS-CoV-2. This was in agreement with the effect of STAT1 in a mouse SARS-CoV infection model (91). Surprisingly, the severe lung damage presented in SARS-CoV-2 infected WT hamsters was absent in STAT2 KO hamsters, contrary to general observations of viral infections in STAT2−/− mice (92) or STAT2−/− hamsters (93–95). Indeed, pneumonia was absent in SARS-CoV-2 infected STAT2−/− hamsters. Accordingly, this points to an important role of STAT2 in systemic inflammation and severe COVID-19 and identifies it as a novel therapeutic target. Moreover, hamsters could serve as a preferred infection model for the preclinical assessment of vaccines or antiviral therapies, and of anti-inflammatory strategies controlling the hyperactive immune response in severe COVID-19 (96, 97).

## Systemic Inflammation and multiple Organ Damage in Severe COVID-19

As shown above, SARS-CoV-2 mainly infects the upper and lower respiratory system but also targets extrapulmonary organs. An important cause of death in COVID-19 disease is ARDS, which is characterized by the “cytokine storm”. This results from the release of pro-inflammatory cytokines and chemokines and IFNs by immune effector cells, which mediates widespread inflammation and damage to other vulnerable organs. This can rapidly progress to multiple organ damage, which correlates with a poor prognosis in COVID-19 patients. So far several molecular mechanisms have been described that cause widespread inflammation and damage to vulnerable organs, including cardiovascular, renal, and neurological systems, and contribute to multiple organ exhaustion and death in COVID-19.

### Cardiovascular System

With the growing number of COVID-19 infected cases and accumulating clinical data, COVID-19 related cardiovascular diseases have attracted a great deal of concern over an associated mortality rise among infected patients. Likewise, the strong correlation of risk factors such as hypertension and cardiovascular disease with the severity of COVID-19 outcome has already been well established (98). In this aspect, the cardiovascular manifestations in patients with COVID-19 encompass a wide range of complications including myocardial infarction, arrhythmias, heart failure, and hypercoagulation (99). It is well known that ACE2 is abundant in the heart, especially in pericytes, coronary endothelial cells, cardiomyocytes, and cardiac fibroblasts (100). Following SARS-CoV-2 infection and downregulation of ACE2, RAAS is over-activated and may contribute to thrombotic events and inflammatory responses and ultimately leading to microvascular dysfunction and injury (101). Coagulation abnormalities are evidently seen in nearly 20%–55% of hospitalized patients with COVID-19 (102). For instance, in a cohort study of confirmed COVID-19 patients, the elevated levels of D-dimer and fibrin degradation product (FDP) along with prolonged prothrombin time and activated partial thromboplastin time were frequently seen in COVID-19 non-survivors (103). This observation indicates the high incidence of thromboembolic events in COVID-19 cases which might impact vascular homeostasis (104).

In a recent systemic review investigating the potential contribution of hyper-inflammation in the induction of cardiac injuries in the COVID-19 context, the authors concluded that systematic hyper-inflammatory response may trigger cardiac injury in COVID-19 patients including children and adolescents without having pre-existing cardiovascular abnormalities (105). For the hyperinflammation assessment, the markers C-reactive protein(CRP) and/or ferritin and/or IL-6 were employed in their investigation, as both ferritin and IL-6 have been previously shown increases in severe COVID-19 cases in contrast to survivors (106). A retrospective study that explored the relationship between SARS-CoV-2-induced hyperinflammatory response in 317 patients with COVID-19, reported that 14 out of 39 patients with a myocardial injury who showed immune dysregulation, developed myocardial injury nine days after admission (107). This finding may imply that cardiac injury is elicited by the development of inflammatory storms in severe COVID-19 cases. In other words, cytokine storms may contribute to apoptosis or necrosis of the myocardial cells, compromising the hemodynamics of coronary circulation, destabilizing coronary plaque, and instigating vascular microthrombosis (108).

### Urinary System

Kidney injury in a quarter of hospitalized patients with COVID-19 has been described in several studies. Indeed, COVID-19 associated acute kidney injury (AKI) is reported with a high mortality rate, particularly in critically ill patients with comorbidities such as hypertension and diabetes (109). Urine analysis of COVID-19 patients displayed alterations in urine sediments including proteinuria and hematuria which is linked with elevated mortality in hospitalized COVID-19 patients (110). Moreover, adequate evidence for the presence of SARS-CoV-2 RNA in the urine of COVID-19-positive patients does not seem robust to accurately predict renal dysfunction (111).

The pathophysiological mechanisms underlying COVID-19 associated AKI remains to be clarified. However, there are COVID-19-specific mechanisms that might play a role in renal dysfunction. Among all, the relationship between ACE2 and the renin-angiotensin-aldosterone system (RAAS) which is disturbed by COVID-19 infection is regarded as a major mechanism engaged in kidney injury (112). In fact, ACE2 as the main receptor for SARS-CoV-2 and highly expressed in kidney (113), is able to convert angiotensin II to angiotensin 1–7, down-regulating RAAS-mediated platelet and endothelial activation, vasoconstriction and release of proinflammatory mediators. However, upon binding of SARS-CoV-2 to ACE2, followed by accumulation of angiotensin II and overactivation of RAAS, leading subsequently to hypercoagulability, endothelial injury, and inflammatory response (114). Based on this understanding, drugs that inhibit RAAS have been suggested as a potential treatment for COVID-19 (115), but it still demands more tailor-made prospective randomized controlled studies to confirm the efficacy of these drugs.

Cytokine storm syndrome which is portrayed as a hyperactive state of the immune system through extensive recruitment of immune cells, is believed to substantially contribute to the severity of COVID-19 infection (116). Likewise, upregulation of cytokines namely IL-6 which is a key element in multi-organ damage including kidney injury, has been frequently recorded in COVID-19 patients (117). It was shown that IFN-α and IFN-β display mutual and differential effects on podocytes and parietal epithelial cells, which collectively advance glomerulosclerosis by inducing podocyte loss and also restricting podocyte regeneration from local progenitors (118). This is of considerable importance since inflammation-induced proteinuria is mediated by podocyte injury and aberrant type I interferon response can further aggravate proteinuric kidney disease (119). Moreover, a growing body of evidence showed the renal levels of C5b-9 which engaged in the complement membrane attack complex, were elevated in severe COVID-19 patients compared with healthy controls. Similarly, C5a triggers abnormal epigenetic changes in renal tubular epithelial cells causing tubular senescence. In addition, C5b–9 is involved in endotheliopathy and thromboinflammation (117, 120). In light of these findings, complement activation might drive inflammation associated with coagulation and thrombosis in the context of COVID-19, exacerbating kidney dysfunction towards to progression of AKI.

### Nervous System

There is also a growing body of evidence pointing to complications of the nervous system in patients with COVID-19. These neurological and neuropsychiatric symptoms range from smell and taste alterations to infectious encephalitis. The underlying mechanisms involved in these neurological manifestations are not entirely understood. However, it seems systemic inflammation and deregulated immune activity might cast new light on the causes of SARS‐CoV‐2-associated neuropathogenesis.

There are conflicting reports about the direct invasion of SARS‐CoV‐2 to the brain (121–123). Indeed, the varied expression levels of SARS-CoV-2 entry receptors in the central nervous system propose potential routes including the olfactory system and blood-brain barrier (BBB) for direct viral infection. For instance, it was shown that ACE2 and TMPRSS2 are expressed in the cells located in the olfactory epithelium, except for olfactory sensory neurons (124). Likewise, the expression of ACE2 is detected in pericytes which is a part of the neurovascular unit in BBB (125).

According to a meta-analysis of postmortem examinations in COVID-19 patients with neurological manifestations, the main neuropathological features that were documented among over 190 cases were gliosis including astrogliosis and microgliosis, hypoxic alterations, infiltration of inflammatory cells such as macrophages and T- lymphocytes, cerebral microthrombosis, ischaemic brain lesions. In light of these observations, it seems that systemic inflammation and dysregulated coagulation substantially drive neuropathological outcomes including hemorrhages and microthrombi in the context of COVID-19 infection, compared with the injuries caused directly by SARS-CoV-2 infection (126). The contribution of gliosis is further supported by the neuropathological examinations of 43 patients with COVID-19, in which the interplay between activated immune response and nervous system was of considerable note in all examined brain regions where reactive astrogliosis was detectable at varying levels of intensity (122). In fact, reactive astrocytes are regarded to be an indispensable part of the neuroimmune system by promoting neuroprotection in infectious diseases. However, following SARS-CoV-2 infection, the expression levels of glial fibrillary acidic protein (GFAP), as an astrogliosis marker, are highly induced in patients with moderate to severe COVID-19 (127, 128), suggesting the possible role of reactive astrocytes along with aberrant microglial functions in shaping COVID-19-induced cytokine storm, leading ultimately to multiple-organ failure.

Current research on dysregulated immune activity in COVID-19 shows the upregulation of circulating cytokines such as TNF-α, IL-6, and IL-10 (4, 129), suggesting peripheral inflammation induced by COVID-19 might be disseminated to brain parenchyma through compromised BBB and severely affect neurons and glial cells. In this regard, we recently showed the increased levels of neuroinflammatory genes along with gliosis and neuronal death in the cerebral cortex of COVID-19 patients (127), as also reported in (130). Additionally in our study, upon the close histopathologic examination of the post-mortem cerebral cortex of patients with COVID-19, inflammation of blood vessels (vasculitis) was detected, which might pave the way for lymphocytic infiltration and neuronal damage, as already documented in a case report of COVID-19 (131). Thus, disrupted BBB undermines the regulation of BBB permeability and makes the brain vulnerable to the invasion of pathogens and entry of pro-inflammatory mediators, leading to severe systemic disease.

## STATs as Potential Therapeutic Targets in COVID-19 Patients

Implications for recombinant IFN therapy have strictly been proposed in the early stages of COVID-19 disease, especially during upper respiratory tract infection of SARS-CoV-2. In this respect, the kinetics of IFN-I secretion versus the kinetics of viral replication should help in identifying the window of therapeutic opportunity.

On the other hand, significant progress has been made on therapeutic approaches that can manage the systemic inflammation and hyperactive immune response in severe COVID-19 patients (29, 132–134). Currently, at clinicaltrials.gov over one hundred clinical trials are registered connected to inflammatory cytokine blocking medications as potential treatment strategy in COVID-19 patients.

As the JAK/STAT pathway modulates important inflammatory and immunological pathways, JAKs and STATs serve as potential therapeutic targets in severe COVID-19. Anti-IL6 antibodies (Tocilizumab), BTK inhibition with ibrutinib and acalabrutinib, as well as the known JAK inhibitors- Ruxolitinib, Fedratinib, and Baricitinib are part of potential treatment strategies against COVID-19 that combine antiviral and anti-inflammatory agents (135–139). Ruxolitinib entered phase III clinical trials (NCT04120090, NCT03533790) and Fedratinib phase II, both were used in connection with pneumonia-associated COVID-19 disease.

Strategies to inhibit the JAK-STAT pathway identified numerous JAK inhibitors (Jakinibs), which mostly are known as pan-JAK inhibitors. For example, the FDA approved Tofacitinib, Ruxolitinib, Fedratinib, and Baricitinib, which inhibit multiple JAKs. Likewise, a high number of small molecules have been identified that target the pTyr-SH2 interaction area of STAT3, and in analogy to Jakinhibs indicated as Statinibs (140, 141). They were recently compiled by our group in SINBAD, a curated open-access database of more than 175 Statinhibs which have been published and described in scientific articles providing proof of their inhibitory properties. It is a tool allowing user-friendly analysis of experimental conditions and provides detailed information about known STAT inhibitory compounds (142); datasets are available at http://sinbad.amu.edu.pl as well as through the public repository https://doi.org/10.6084/m9.figshare.14975136.v1 (142).

Among the first reported Statinhibs was STA-21, which was shown to inhibit STAT3 dimerization, DNA binding, and STAT3-dependent transcription in breast cancer cells (143). Similarly, the known Statinhibs STATTIC and STX-0119 inhibited phosphorylation, dimerization and nuclear translocation of STAT3, leading to increased apoptosis of cancer cells [reviewed in (87, 144, 145)].

As part of a novel strategy to characterize and understand STAT-inhibition, we combined comparative *in silico* docking and virtual screening of multiple STAT-SH2 models with *in vitro* validation of STAT inhibition (146, 147). This led to the identification of a novel class of multi-STAT or pan-STAT inhibitor, C01L_F03 that commonly and with equal affinity targets the SH2 domain of STAT1, 2, and 3. Interestingly, STATTIC and STX-0119 displayed similar STAT cross-binding characteristics. During anti-microbial and inflammatory responses, macrophage activation, and cytokine release is tightly regulated by IFN and TLR signaling. Accordingly, maximum expression of important pro-inflammatory and chemokine genes depends on cross-talk between IFNs and TLR signaling through collaborative actions of STATs, IRFs, and NF-kB. Recently, we further proved that in macrophages, dendritic cells and vascular smooth muscle cells, under conditions of IFN and TLR cross-talk, the above-mentioned novel pan-STAT inhibitors (with STATTIC being the most potent one) managed genome-wide inhibition of multiple cytokines, chemokines and anti-viral products. Accordingly, a novel STAT-inhibitory strategy was developed for the inhibition of pro-inflammatory cytokine-dependent migration of endothelial cells, adhesion of leukocytes to endothelial cells, and loss of contractility of mesenteric arteries (148).

Promising results for several FDA-approved inhibitors [Tofacitinib: pan-JAK inhibition; Ruxolitinib: JAK1/2-inhibition; Tocilizumab: IL6 receptor antibody); or (pre)Clinical Trial tested [sb1578: JAK2-inhibition; WP1066: JAK2-inhibition; STA-21: STAT3-SH2 inhibition; STATTIC: STAT3-SH2 inhibition], promises entry of Statinhibs to the clinic shortly (87). So far, Statinhibs have not been implicated in connection to COVID-19. However, the further testing and optimizing of already available Statinhibs, together with the identification of new Statinhibs, may offer new clinical benefits in combating immune hyperactivation during severe COVID-19. In this context, according to Toubiana et al., “care must be taken to apply STAT targeting therapeutic strategies in a stage-appropriate manner, because some approaches that may help in the earlier stages of the disease could be detrimental if used in its later stages. With the potential use for COVID-19 treatment of existing drugs that enhance STAT1 or inhibit STAT3 functions, problems could arise. For example, chronic mucocutaneous candidiasis (CMC) was present in 98% of patients with gain of function (GOF) mutations in STAT1 in one study of 274 individuals, and the few that did not have CMC, had invasive fungal or bacterial infections. Generally, the immune profile was relatively normal, but 82% had decreased Th17 cells” (149). Conversely, a STAT-dependent pro-inflammatory gene signature could serve as a novel diagnostic tool to monitor and diagnose IFN responses, disease progression, and responses to treatment using blood or nasal swabs from COVID-19 patients (**Figure 4**).

## Author Contributions

MEB was involved in concept development and writing and editing of the manuscript. AS, AA and SH designed figures. MS, HN, NL, AO, KK, and JW participated in the development of the concept and critically evaluated and edited the manuscript. HB developed the concept and was involved in writing and editing the manuscript and coordinated input from all co-authors. All authors contributed to the article and approved the submitted version.

## Funding

This publication was supported by grant UMO 2016/23/B/NZ2/00623 (HAR.B.) from the National Science Centre Poland and grant 7/2020 (HAR.B.) from the Adam Mickiewicz University in Poznan.

## Conflict of Interest

The authors declare that the research was conducted in the absence of any commercial or financial relationships that could be construed as a potential conflict of interest.

## Publisher’s Note

All claims expressed in this article are solely those of the authors and do not necessarily represent those of their affiliated organizations, or those of the publisher, the editors and the reviewers. Any product that may be evaluated in this article, or claim that may be made by its manufacturer, is not guaranteed or endorsed by the publisher.
